# Identification of non-invasive biomarkers for chronic atrophic gastritis from serum exosomal microRNAs

**DOI:** 10.1186/s12885-019-5328-7

**Published:** 2019-02-08

**Authors:** Hong Liu, Pei-wu Li, Wei-qin Yang, Hong Mi, Jing-lin Pan, Yuan-cheng Huang, Zheng-kun Hou, Qiu-ke Hou, Qi Luo, Feng-bin Liu

**Affiliations:** 1 The First Affiliated Hospital of Guangdong Pharmaceutical University, No.19 Nonglinxia Road, Guangzhou, 510080 Guangdong Province China; 2 Guangzhou University of Chinese Medicine, No.12 Jichang Road, Guangzhou, 510405 Guangdong Province China; 3 The Eight Affiliated Hospital, Sun Yat-sen University, No.3025 Shennanzhong Road, Shenzhen, 518033 Guangdong Province China; 4 The First Affiliated Hospital of Guangzhou University of Chinese Medicine, No.16 Jichang Road, Guangzhou, 510405 Guangdong Province China; 5 Lingnan Medical Research Center of Guangzhou University of Chinese Medicine, No.12 Jichang Road, Guangzhou, 510405 Guangdong Province China

**Keywords:** Biomarkers, Serum exosomes, microRNAs, Small RNA-sequencing, Chronic atrophic gastritis

## Abstract

**Background:**

Serum exosomal microRNAs (miRNAs) have been suggested as novel biomarkers for various diseases, especially gastric cancer (GC). But circulating biomarkers for Chronic atrophic gastritis (CAG) which is defined as precancrerous lesions of GC remain largely elusive. To investigate serum exosomal miRNAs that are differently expressed in CAG patients and Chronic nonatrophic gastritis (CNAG) may be helpful for its diagnosis and therapy.

**Methods:**

Patients were recruited according to the diagnosis and exclusioncriteria. RNA was extracted from serum exosomes of 30 CAG and 30 CNAG patients. The miRNA expression profiles were analyzed by next generation sequencing and were validated by qRT-PCR. Receiver operating characteristic (ROC) analysis has been used to evaluate the diagnostic value.

**Results:**

30 CAG patients and 30 CNAG patients were recruited in our study. sRNA-seq results showed that hsa-miR-3591-3p, − 122-3p, and − 122-5p of the top 10 miRNAs (hsa-miR-148a-3p, − 122-3p, − 486-3p, −451a, − 122-5p, − 3591-3p, − 486-5p, −151a-3p, −92a-3p, −320a) were significantly upregulated in exosomes from CAG patients versus those from CNAG patients, but hsa-miR-451a, −151a-3p, and -92a-3p were significantly downregulated. Furthermore, qRT-PCR analysis confirmed that hsa-miR-122-5p and hsa-miR-122-3p were significantly upregulated in CAG samples, but hsa-miR-122-3p hadnot a steable expression. ROC curves showed that the AUC for hsa-miR-122-5p was 0.67 (95% CI 0.52–0.82, SE 62%, SP 86%). A sum of the four miRNAs (panel 1, hsa-miR-122-5p, −451a, −151a-3p, and -92a-3p) did not significantly improve the diagnostic potential (AUC 0.63, 95% CI 0.47 to 0.78). Correlation analysis showed that the expression of hsa-miR-122-5p differed significantly between patients based on atrophic (Moderate atrophic vs. Absent, *P* value was 0.036.) and IM (compare moderate-severe, absent and mild *P* values were 0.001 and 0.014, respectively). However, there were no differences between groups based on age, gender, dysplasia, or chronic or active inflammation.

**Conclusion:**

These results suggested that hsa-miR-122-5p in serum exosomes might serve as a potential biomarker for CAG diagnosis.

**Trial registration:**

Chinese Clinical Trial Registy (ChiCTR-IOR-16008027, Date of Registration:2016-03-01).

**Electronic supplementary material:**

The online version of this article (10.1186/s12885-019-5328-7) contains supplementary material, which is available to authorized users.

## Background

Chronic atrophic gastritis (CAG) is one of precancerous stage of intestinal type gastric cancer (GC) and has a high prevalence [1]. CAG also usually progresses to intestinal metaplasia (IM) and dysplasia [2]. Over the last 5 years, the annual incidence of GC was found to be 0.5–1% for CAG, 0.6% for mild-to-moderate dysplasia, and 6% for severe dysplasia [3, 4]. The pathogenesis of CAG remains as a complicated context, at least including immune-mediated inflammation, atrophy of mucosal gland, activated serum auto-antibody for gastric parietal cells and/or intrinsic factors, hypochlorhydria, deficiency of vitamin B12 and, in some cases, neurological symptoms and diffuse metaplasia [5]. Additionally, the diagnosis and follow-up examination of CAG depends on endoscopic examination and histopathologic evaluation, which are invasive and time-consuming for patients [1]. There should be biopsies taken from more sites to avoid misdiagnoses. Even so, gastrin-17(G-17) and Pepsinogen (PG) I or II may be helpful for determining atrophy existence and location. But it had not been widely used with low stability cases [6, 7]. Thus, it is urgent to investigate some non-invasive, sensitive and reliable biomarkers, which may enrich method of CAG diagnosis.

Biomarkers that can be measured in biofluids, called as liquid biopsies [8]. It is more comfortable than endoscopic examination and may reflect the heterogeneity of the disease. Remarkably, exosomes, a new type of liquid biopsy, may show the disease status [9, 10]. Recently, more and more studies have reported that miRNAs are secreted from different types of cells through exsomes (30–100 nm) or other extracellular vesicles (EVs) such as microvesicles (100–1000 nm) and oncosomes (1–10 μm) [9, 11]. They play important roles in cell-to-cell communication, such as in inflammatory response as well as in tissue regeneration and tumor progression [12]. Serum exosomal miRNAs can be potential biomarkers for various diseases. For example, serum exosomal miR-19b-3p and miR-106a-5p have been identified as gastric cancer biomarkers [13]. However, reports of the expression of serum exosomal miRNAs in CAG have not been found.

In the present study, small RNA-sequencing (sRNA-Seq) was employed to analyze microRNA profiles in serum exosomes of CAG patients. qRT-PCR and correlation analyses were used to validate the results of sRNA-seq. We tried to find some specific biomarkers for CAG progression. So far as we know, It might be the first report about serum exosomal miRNAs and potential biomarkers associated with CAG. Our study provides new avenues and references for determining biomarkers and elucidating the underlying mechanisms of CAG.

## Methods

### Patients and samples

Two groups of patients were recruited from 2016 to 2017 at the First Affiliated Hospital of Guangzhou University of Chinese Medicine in Guangzhou, China. Patients were required to provide medical history, to receive physical examinations and laboratory safety tests and to undergo a gastroscopy and biopsies for histological investigation. CAG group was composed of patients who had a diagnosis of CAG with or without intestinal metaplasia, and Chronic non-atrophic gastritis (CNAG) group is composed of health honors and patients who had mild or moderate superficial gastritis. Patients considered to be eligible subjects were between 18 and 70 years old [14]. Exclusion criteria included severe dysplasia, suspicion of gastric cancer or any other malignancy, gastric surgery history, drug allergy to the studied medication, severe systemic diseases (cardio-cerebral-vascular disease, hepatic diseases, blood, kidney, lung or liver disease), administration of non-steroidal anti-inflammatory drugs, pregnancy or lactation in female patients, and unwillingness to undergo repeated endoscopy after treatment.

The study was approved by the Ethics Committee of the First Affiliated Hospital of Guangzhou University of Chinese Medicine, and it was one part of our trial study (ChiCTR-IOR-16008027). All participants provided informed consent and were required to have a negative reports of 13C-urea from a breath test to show that they were not infected with H.pylori. Clinical and demographic information was obtained from each study subject using a combination of structured subject interviews and medical records. Blood (10 ml) and gastric mucosal biopsies were collected from each patient. Serum was separated immediately and stored at − 80 °C before use.

### Exosomes isolation

Exosomes were isolated from serum using ExoQuick™ (SBI, USA). Serum was centrifuged at 17,000 g for 10 min and filtrated with a 0.22 μm syringe filter (Millipore, USA) to remove cell debris. Then 500 μL of serum supernatant was moved into a new tube and mixed with 125 μL ExoQuick reagent. After incubation at 4 °C overnight, the mixture was centrifuged at 1500 g for 30 min. The pellet containing exosome fractions was then resuspended in 30-40 μL of PBS or used for the next analysis.

### Exosomes characterization

Exosomes were characterized with NanoSight analysis and flow cytometry. The size distribution of the exosomes was analyzed using the ZETASIZER Nano series-Nano-ZS (Malvern Instruments Ltd., UK) according to the manufacturer’s instructions. The exosome markers, CD81 and CD63, were identified using flow cytometry. The exosome pellet was resuspended in 100 μL sterile PBS, then incubated with CD63-Antibody-FITC (BD557288, USA) and CD81-Antibody-FITC (BD551108, USA) overnight at 4 °C. Finally, detection was performed using an Accuri C6 flow cytometer (BD Instruments Ltd., USA).

### Exosomal RNA isolation

Before RNA was isolated, samples were treated with proteinase K and RNase A to degrade potential RNA/protein complexes. Exosomal RNA (exo-RNA) was isolated from exosome fractions using a SeraMir Exosome RNA Purification Column kit (SBI, USA), with a final elution volume of 30 μl, according to the manufacturer’s instructions. After RNA isolation, the samples were treated with DNase to degrade potential DNA. The integrities and concentrations of the RNA samples were determined photometrically at 260 nm and 280 nm on a NanoDrop (PeqLab, Germany), then 500 ng or 10 ng of exosomal miRNAs was subjected to ultra-deep sequencing or qRT-PCR.

### Small RNA library preparation and sequencing

30 RNA samples of each group were randomly allocated into three sample pools. Small RNA deep sequencing (SRNA-seq) and bioinformatic analyses were used for the construction of cDNA libraries. Cluster generation and subsequent ultra-deep sequencing using the Illumina platform were performed at Genomics Institute Tech (BGI, China). BGI was routinely used in the bioinformatics analysis process. Briefly, the 49 nt sequence tags from Hiseq sequencing went through the data cleaning analysis first, which included eliminating the low quality tags and 5′ adaptor contaminants from the 50 nt tags, in order to get credible clean tags. Subsequently, the distribution lengths of the clean tags as well as those of the common and specific sequences between samples were summarized. During standard analysis, the clean tags were allocated into different categories, and those which could not be allocated into any category were used to predict the novel miRNA and the seed edit of potential known miRNAs. The fragments of rRNA, scRNA, snoRNA, snRNA and tRNA were removed.

### miRNAs verification by quantitative real-time PCR (qRT-PCR)

qRT-PCR assays were performed using the Mir-X™ miRNA First-Strand Synthesis Kit and the SYBR® qRT-PCR Kit (Takara, Japan). The specific primers for miRNAs were synthesized by Sangon Biotech Company (Shanghai, China). The PCR reaction mixture contained 2 μL of first-strand cDNA, 10 μL of the 2× SYBR advantage Premix, 0.5 μL of the MRQ 3’Primer, 0.5 μL of the miRNA-specific primer (10 μM), and 7 μL ddH2O, having a final volume of 20 μL. After initial denaturation at 95 °C for 10 s, cycling was performed as follows: 95 °C for 5 s and 60 °C for 20 s for 45 cycles in CFX96TM Real-Time PCR Detection System (BioRAD, USA). The qRT-PCR for each miRNA was repeated three times. The target sequences (5′-3′) of the primer sets are shown in Table S1. U6 snRNA was used for normalization, as it has previously been used for normalization in other studies [15]. miRNAs with Cq values above 37 were considered as not having been expressed.

### Statistical analysis

The sRNA-seq data were analyzed in J-Express 2011. DEGseq was used to detect and visualize the intensity-dependent ratios of sRNA-seq data. The miRNAs with more than a 1.2-fold change or less than a 0.6-fold change and a Q value of 0.01 compared to a normal control were considered to be differentially expressing miRNAs (DEMs). For analysis of qRT-PCR data, relative miRNA expressions were calculated using the 2^-ΔCT^ method (ΔCT = CTmiR - CTreference). The 2^-ΔCT^ dataset was then Log2-transformed when necessary. Statistical analysis was executed using a t-test or a Mann-Whitney U test. The area under the curve (AUC) used to assess the diagnostic power of the predictors was determined using Prism V (Graphpad Software). Logistic regression was used to develop a panel of combined biomarkers to predict the probability of developing CAG, as previously described [16]. Correlation analysis was conducted to evaluate whether the miRNA expression levels were correlated with clinical characteristics. *P <* 0.05 was considered significant.

## Results

### Clinical characteristics of patients

30 CNAG and 30 CAG patients participated in our study (The clinical characteristics showed in Additional file 1: Table S2). The ages*(Mean ± SD,* years old*)* in CNAG and CAG groups were 48.67±9.12 years old and 52.67±9.74, respectively. The Male:Female ratios in CNAG and CAG groups were 14:16 and 12:18, respectively. There were no differences in age or gender between CNAG and CAG groups (*P* > 0.05). The two groups were comparable.

### Isolation and identification of exosomes

**Fig. 1:**
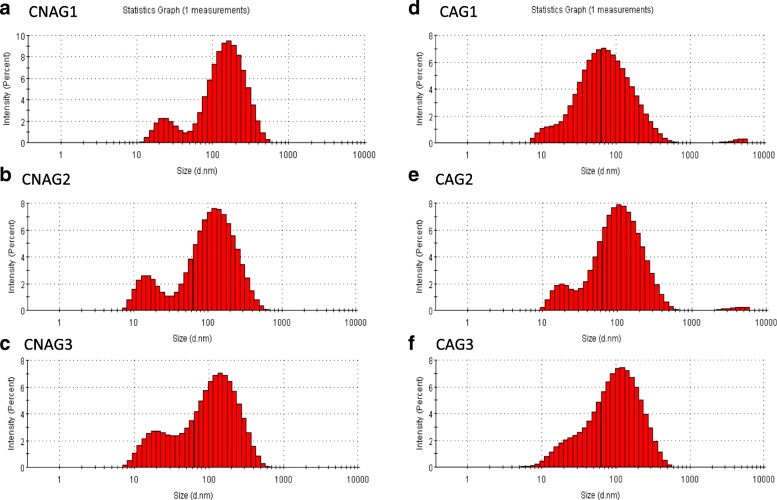
NanoSight analysis for serum exosomes in PS patients. Horizontal axis, particle size (nm); vertical axis, intensity (percent). **a**-**c**: size distribution of isolated exosomes in three donors of CNAG group; **d**-**f**: size distribution of isolated exosomes in three donors of CAG group

**Fig. 2:**
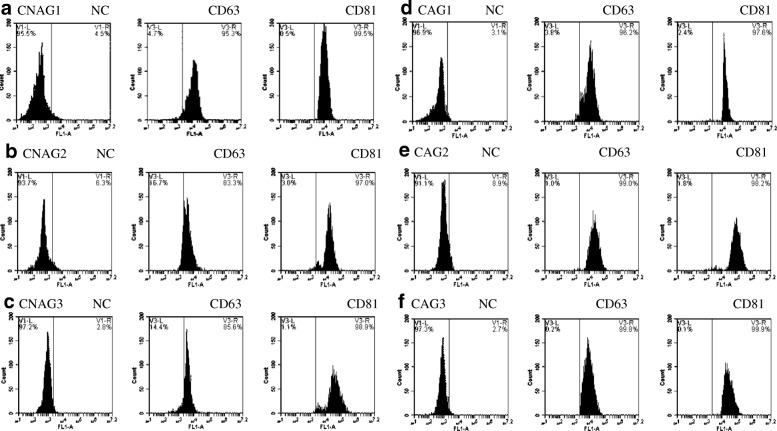
Flow cytometry analysis using exosome markers CD81 and CD63 antibodies of serum exosomes coupled to 0.4 μm beads. Negative control (NC) corresponds to secondary conjugated antibody. **a**-**c**: the expression of exosome markers CD81 and CD63 in three donors'isolated exosomes of CNAG group; **d**-**f**: the expression of exosome markers CD81 and CD63 in three donors'isolated exosomes of CAG group

NanoSight analysis revealed that the size distribution of our isolated exosomes ranged from approximately 20 nm to 200 nm in diameter, with a mode value of 100 nm (Fig. 1). Flow cytometry analysis demonstrated that the expressions of CD81 and CD63, i.e., the commonly used exosome markers, in each sample were over 80% (Fig. 2) [17, 18]. So we concluded that our samples were enriched in exosomes and co-isolated with other extracellular vesicles to some extent.

### Analysis for sRNA-seq data

**Fig. 3:**
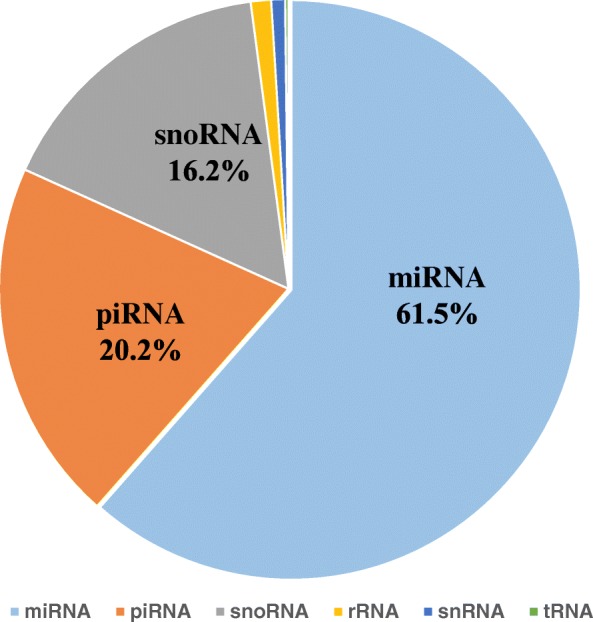
Pie chart showing the percentage of different kinds of sRNA to the mapped reads

Three sample pools of each group were used for sRNA-seq. The results showed that an average of 3.7 million raw reads per sample pool were obtained and that approximately 50.2% of the reads were mapped to the genome (Additional file 1: Table S3, Fig. 3). In the mapped reads, they were mainly to miRNA (61.5%) but also to piRNA (20.2%), snoRNA (16.2%), and the 2.1% rest were mapped to rRNA, snRNA and tRNA. The miRNA read counts, normalized to Transcripts Per Million (TPM), were used in further analyses.

The top 10 most highly expressed miRNAs were hsa-miR-148a-3p, − 122-3p, − 486-3p, −451a, − 122-5p, − 3591-3p, − 486-5p, −151a-3p, −92a-3p and -320a. MiRNAs with less than 10 Readnum per sample were filtered out, leaving 220 miRNAs (Additional file: Table S4), concluding 7 novel miRNAs (novel_mir607, novel_mir363, novel_mir1399, novel_mir503, novel_mir780, novel_mir53, novel_mir1) or 111 miRNAs, if miRNAs from different precursors were considered together.

**Fig. 4:**
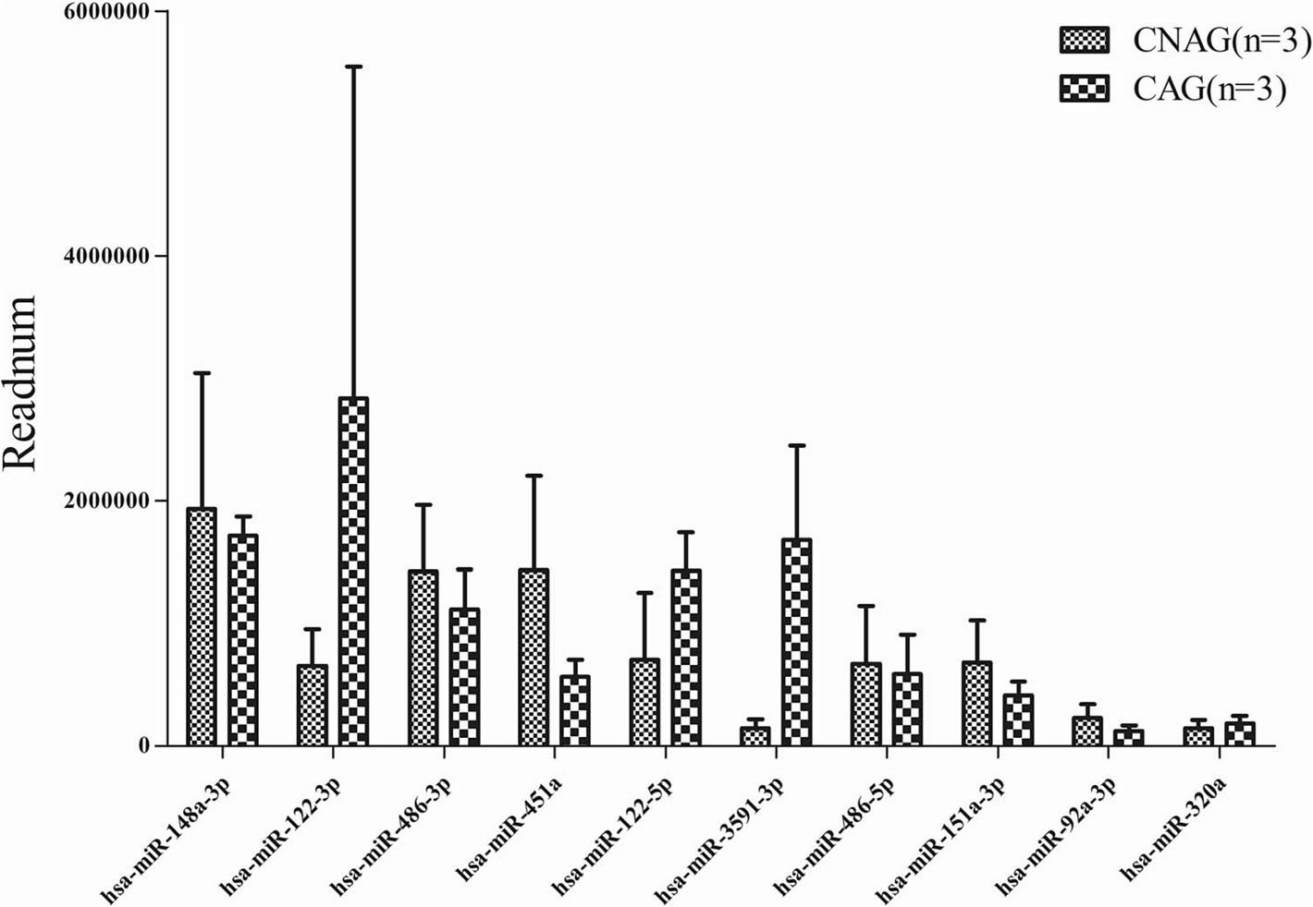
Top 10 most highly expressed miRNAs in serum exosomes

According to the DEGseq, hsa-miR-3591-3p, − 122-3p and − 122-5p were significantly upregulated, and hsa-miR-451a, −151a-3p and -92a-3p were significantly down-regulated in serum exosomes of CAG patients, compared to those of CNAG patients. As a result, those 6 miRNAs have been defined as miRNA candidates (Table 1, Fig. 4).

**Table 1:** The DEGseq^a^ results of top 10 highly expressed miRNAs

miRNA id	CNAG	CAG	log2Ratio (CAG/CNAG)	Regulation	*P*-value	q-value
hsa-miR-122-3p	1,960,665	8,521,642	1.87^b^	UP	< 0.0001	< 0.0001
hsa-miR-122-5p	2,114,731	4,290,194	0.77^b^	UP	< 0.0001	< 0.0001
hsa-miR-148a-3p	5,804,498	5,150,184	− 0.42	DOWN	< 0.0001	< 0.0001
hsa-miR-151a-3p	2,045,393	1,239,824	−0.97^b^	DOWN	< 0.0001	< 0.0001
hsa-miR-320a	435,750	552,718	0.10	UP	< 0.0001	< 0.00010
hsa-miR-3591-3p	436,401	5,052,588	3.29^b^	UP	< 0.0001	< 0.0001
hsa-miR-451a	4,299,557	1,698,145	−1.59^b^	DOWN	< 0.0001	< 0.0001
hsa-miR-486-3p	4,269,795	3,349,858	−0.60	DOWN	< 0.0001	< 0.0001
hsa-miR-486-5p	2,010,115	1,756,101	−0.44	DOWN	< 0.0001	< 0.0001
hsa-miR-92a-3p	688,245	369,669	−1.14^b^	DOWN	< 0.0001	< 0.0001

(^a^Both SAM and rank product wfere used as statistical analysis. ^b^ > 1.2 fold change or < 0.6 fold change and a Q value 0.01 compared with CNAG)

### Validation of sRNA-seq data by qRT-PCR analysis

**Fig. 5:**
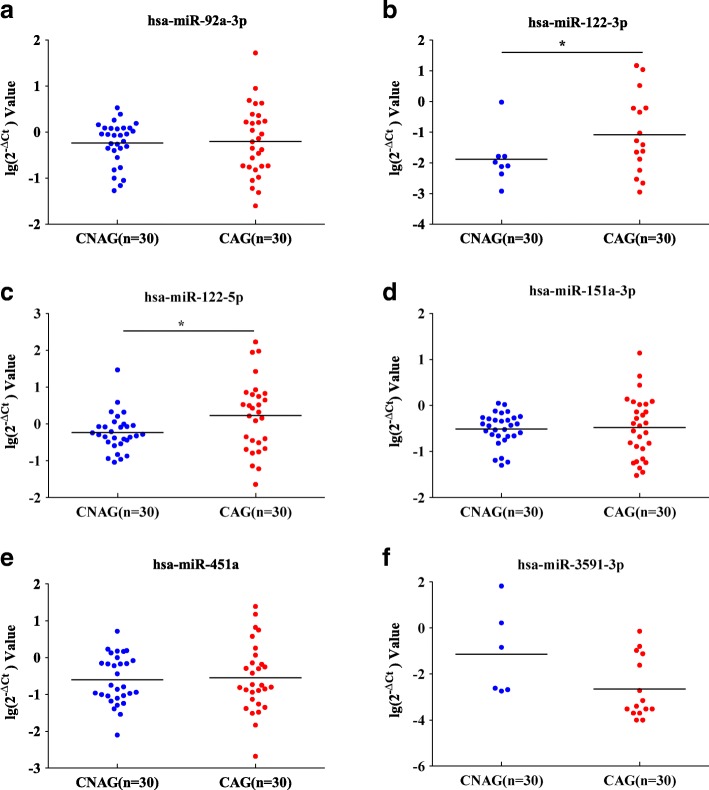
The qRT-PCR analysis of miRNAs. **a**: hsa-miR-92a-3p; **b**: − 122-3p; **c**: − 122-5p; **d**: -151a-3p; **e**: -451a and **f**: − 3591-3p were analyzed in CAG patients and CNAGs. *P < 0.05 versus CNAG group

qRT-PCR analysis was performed for each sample to validate 6 miRNA candidates. The results showed that hsa-miR-122-3p and − 122-5p were significantly upregulated in the CAG group and that they changed consistently with sRNA-seq (Table 2, Fig. 5). There were no significant differences in the levels of hsa-miR-92a-3p, −151a-3p, miR-451a and − 3591-3p between two groups. However, hsa-miR-3591-3p and hsa-miR-122-3p were not detected in every sample, which may be due to their poor expression. Therefore, hsa-miR-122-5p appears to be the most promising biomarker in CAG patients.

**Table 2:** The qRT-PCR results (log2^-△ct^ value) of miRNAs

Group	hsa-miR-92a-3p	hsa-miR-122-3p	hsa-miR-122-5p	hsa-miR-151a-3p	hsa-miR-451a	hsa-miR-3591-3p
CNAG	−0.24 ± 0.46	−1.97 ± 0.82	−0.23 ± 0.51	−0.51 ± 0.35	−0.60 ± 0.66	−1.14 ± 1.89
CAG	−0.20 ± 0.75	−0.98 ± 1.26	0.23 ± 0.98	−0.48 ± 0.66	−0.54 ± 0.93	−2.67 ± 1.33
t	−0.20	−2.10	− 2.24	− 0.23	−0.27	2.10
P	0.84	0.04*	0.03*	0.82	0.79	0.06

*P < 0.05 versus CNAG group

### ROC analysis of miRNAs

ROC curves for these miRNAs, except those of hsa-miR-122-3p and − 3591-3p, showed that the AUC for hsa-miR-122-5p was 0.67 (95% CI 0.52–0.82, SE 62%, SP 86%) (Table 3). A sum of the four miRNAs (panel 1) did not significantly improve the diagnostic potential (AUC 0.63, 95% CI 0.47 to 0.78). Therefore, hsa-miR-122-5p may be helpful for the diagnosis of CAG.

**Table 3:** ROC curves for those miRNAs

miRNAs	AUC	STD	P value	95%CI		SE(%)	SP(%)
hsa-miR-92a-3p	0.51	0.08	0.94	0.35	0.66	50.00	70.00
hsa-miR-122-5p	0.67	0.08	0.03*	0.52	0.82	62.07	86.21
hsa-miR-151a-3p	0.51	0.08	0.93	0.35	0.66	27.59	96.67
hsa-miR-451a	0.53	0.08	0.74	0.37	0.68	77.78	40.00
panel 1^a^	0.63	0.08	0.10	0.47	0.78	46.43	93.33

^a^panel 1 was the sum of hsa-miR-92a-3p, − 122-5p, −151a-3p, −451a. *P < 0.05

### Correlation analysis between hsa-miR-122-5p and clinicopathologic factors

The results demonstrated that the expression of hsa-miR-122-5p was significantly differs between patients, based on atrophic (Moderate atrophic vs. Absent, *P* value was 0.036.) and IM (comparing moderate-severe, absent and mild *P* values were 0.001, 0.014, respectively) values. There were no differences among groups based on age, gender, dysplasia, or chronic inflammation or active inflammation (Table 4). The results showed that the expression of serum exosomal hsa-miR-122-5p might be related to atrophic and IM.

**Table 4:** The expression of serum exosomal hsa-miR-122-5p in groups based on clinicopathologic factors

Variables		hsa-miR-122-5p(lg10)	*P*
		Mean ± SD	
Age	< 50 (*n* = 28)	−0.19 ± 0.74	0.09
	≥50 (*n* = 32)	0.16 ± 0.83	
Gender	Male (*n* = 26)	−0.007 ± 0.76	1.00
	Female (*n* = 34)	−0.005 ± 0.87	
Atrophic	Absent (*n* = 30)	−0.23 ± 0.51	0.01*
	Mild (*n* = 13)	0.22 ± 1.09	
	Moderate (*n* = 11)	0.55 ± 0.74*^a^	
	Severe (*n* = 5)	−0.46 ± 0.93	
Interestnal metaplasia	Absent (*n* = 42)	−0.16 ± 0.67*^b^	< 0.01*
	Mild (*n* = 9)	−0.11 ± 0.77*^b^	
	Moderate-severe (n = 9)	0.79 ± 0.99	
Dysplasia	Absent (*n* = 52)	−0.04 ± 0.83	0.39
	Light-median (*n* = 8)	0.23 ± 0.55	
Chronic inflammation	Mild (*n* = 31)	0.06 ± 0.74	0.68
	Moderate (*n* = 24)	−0.02 ± 0.90	
	Severe (*n* = 5)	−0.24 ± 0.84	
Active inflammation	No activity (*n* = 40)	0.01 ± 0.81	0.97
	Mild (*n* = 17)	−0.05 ± 0.83	
	Moderate (*n* = 3)	−0.02 ± 0.80	

*^a^Moderate atrophic vs. Absent, *P* value was 0.036. *^b^compare Moderate-severe IM, Absent and Mild IM, *P* value were 0.001, 0.014, respectively. *P < 0.05

## Discussion

Up to date, the diagnosis of CAG still depends on endoscopic examination and histopathologic evaluation. Exosomal miRNA played important roles in cell-to-cell communication, tissue regeneration, tumorigenesis and progression [12]. Expression profiles of exosomal miRNA are disease- and tissue-specificity, which were considered as candidate biomarkers [19]. In the present study, six miRNAs had been extremely changed in serum exosomes, particularly hsa-miR-122-5p was significantly upregulated in CAG samples and had the best AUC value among these miRNAs. Moreover, there was a significant correlation between hsa-miR-122-5p expression and atrophic /IM existence. Thus, hsa-miR-122-5p in serum exosomes might serve as a potential biomarker for CAG diagnosis.

Clinically, many volunteers without obvious clinical issues had chronic gastritis more or less when they undergo the gastroscopy. Besides, applying a clinical ethics which includes healthy volunteers with a gastroscopy examination is hardly to be approval by the ethics council or be consented by volunteers themselves. In line with the previous study, CNAG patients who had been diagnosed with normal, mild or moderate superficial gastritis were selected as a control group in the present study [20]. To screen the potential biomarker in CAG, we first compared the expression profiles of miRNA between CAG and CNAG. Our results showed that there was no significant difference in clinical characteristics between the CAG and CNAG, and then we successfully isolated the serum exosomes from CAG and CNAG, Sequencing analysis results firstly considered the abundance of miRNAs to ensure the effective progression of a follow-up study as previous studies [21]. Although there is no report about serum exosomal biomarker for CAG, these studies show that those miRNAs may be involved in the damage, inflammation and even carcinogenesis of gastric mucosa.

In the present study, we first investigated aberrant expression of serum exosomal miRNAs between CAG group and CNAG group. Our results showed that hsa-miR-3591-3p, − 122-3p, − 122-5p were upregulated and hsa-miR-451a, −151a-3p, −92a-3p were down-regulated in CAG. So far as we know, some these following studies related to those miRNAs. Previous studies had showed that hsa-miR-92a was upregulated in GC tissues and was also identified as a secretory miRNA in BGC823 and MGC803 cell lines, which in turn to promotes GC cell growth by targeting E2F1 and HIPK1 [22]. The relationship of miR-451 expression and GC progression remains as a complicated context. MiR-451 was downregulated in gastric cancer, and its proved target were GATA2, ABCB1, MIF [23, 24]. Moreover, miR-451 might regulate macrophage migration inhibitory factor production and the proliferation of gastrointestinal cancer cells [25]. Hsa-miR-3591-3p has been found to be downregulated may through regulate RGS2 in anxiety patients [26]. Therefore, these miRNAs not only served as biomarkers of CAG, but also involved in GC progression.

Then, we confirmed the sRNA-seq results by real-time PCR. Firstly, due to the low abundance of miRNAs in exosomes and the limited clinical samples met our inclusive criteria, in line with the previous study, we only chose U6, the widely used normalizer in exosome studies, to serve as normalizer to economize templates for other target genes amplification [15]. In the present study, most of the isolated miRNAs were prior to perform the RNA-seq, and we upregulated the mount of cDNA for qRT-PCR as much as we could. Our showed that hsa-miR-122-3p and hsa-miR-122-5p were significantly upregulated in CAG group. However, hsa-miR-122-3p and some of the other miRNAs were not detected in every sample. The main reason of these were due to the difference mounts of miRNAs were used in RNA-seq and qRT-PCR, this difference might further increase the variance of results, even reverse them. Therefore, among these miRNA, hsa-miR-122-5p might be the more appropriate biomarker in serum exosomes of CAG patients. The diagnostic value of hsa-miR-122-5p calculated from PCR results was 0.67 (95% CI 0.52–0.82, SE 62.07%, SP 86.21%), and its expression might be correlated with atrophic and IM existence. The discovery of biomarkers may present an alternative method for CAG diagnosis, which are urgently needed. In addition, hsa-miR-122-3p and − 122-5p are mature members of the mir-122 family. MiR-122, known as a hepatic-specific miRNA, is related to the regulation of TAG pathways in liver cells through targeting CTDNEP1/LPIN1 [27–30]. It may could form LINC01296/miR-122/MMP-9 regulation pathways to promote GC cell migration [31]. Remarkably, miR-122 targets NOD2 to decrease intestinal epithelial cell injury in Crohn’s disease [32]. Thus, miR-122 is a powerful miRNA that participates in GC initiation and progression.

Highly expressed hsa-miR-122-5p in CAG serum exosomes may originate from gastric mucosal epithelial cells. Note that levels of exosomal miRNAs do not necessarily represent the cellular levels since the miRNA sorting mechanism may affect the incorporation of miRNAs into exosomes [33]. On the disease level, an increase in the level of a circulating miRNA is probably not the result of an upregulation of the miRNA in the tissues themselves, rather than a negative effect of the disease on the expression of the miRNA in other cells [34]. Further study is needed to explore the role and origin of dysregulated hsa-miR-122-5p in CAG progression. Meanwhile, this should be verified in larger patient cohorts due to the low AUC value; the expression of serum exosomal hsa-miR-122-5p of CAG patients should also be compared with that of gastric cancer patients. It’s not likely to guarantee all of the miRNA markers determined with NGS in the discovery stage could be successfully detected using real-time PCR approaches in the subsequent validation stage. Relatively larger number of miRNA candidates should be seriously considered when a mining of miRNA biomarker moves onto the point of technical transition [35, 36].

In summary, serum exosomal hsa-miR-122-5p appears to be the most promising biomarker for CAG diagnosis, though it remains as a challenge to obtain systematic investigation of the pathological process of CAG when using miR-122-5p. Our study provides new determining serum biomarkers, which may be helpful for the diagnosis of CAG and elucidating the underlying mechanisms of CAG.

## Conclusions

Our study may be the first to identify 6 differentially expressed circulating exosomal microRNAs (hsa-miR-3591-3p, − 122-3p, − 122-5p, −451a, −151a-3p, −92a-3p) in patients with CAG with CNAG, hsa-miR-122-5p may be used as a promising biomarker for diagnosis of CAG.

## Additional files

Additional file 1: **Table S1**. The target sequences of the primer. **Table S2**. Clinical characteristics of patients in this study. **Table S3**. Summary of sequencing data for each sample pool. **Table S4**. Readnum of 220 miRNAs for each sample pool. (DOC 573 kb)

## Abbreviations

AUC: Area under the curve
CAG: Chorinic atrophy gastris
CNAG: Chorinic nonatrophy gastris
EV: Extracellular vesicle
Exo: Exosomes
G-17: Gastrin
GC: Gastric cancer
IM: Intestinal Metaplasia
miRNAs: microRNAs
PG: Pepsinogen
ROC: Receiver operating characteristic
