# Supplementary material for: Identification of non-invasive biomarkers for chronic atrophic gastritis from serum exosomal microRNAs
Source: BMC Cancer. 2019 Feb 8;19:129. doi: 10.1186/s12885-019-5328-7 (PMC6368711; doi:10.1186/s12885-019-5328-7)
Supplement: Supplementary file 1 — Table S1. The target sequences of the primer. Table S2. Clinical characteristics of patients in this study. Table S3. Summary of sequencing data for each sample pool. Table S4. Readnum of 220 miRNAs for each sample pool. (DOC 573 kb) [file 12885_2019_5328_MOESM1_ESM.doc]

Additional file 1:

Table S1. The target sequences of the primer.

| primer | sequences |  | primer | sequences |
| --- | --- | --- | --- | --- |
| hsa-miR-122-3p | gcagaacgccattatcacac |  | hsa-miR-3591-3p | cgcagaaacaccattgtca |
| hsa-miR-451a | cgcagaaaccgttacca |  | hsa-miR-151a-3p | gctagactgaagctccttg |
| hsa-miR-122-5p | gcagtggagtgtgacaatg |  | hsa-miR-92a-3p | cgcagtattgcacttgtc |

Table S2. Clinical characteristics of patients in this study

| Characteristic |  | CNAG(n=30) | CAG(n=30) |
| --- | --- | --- | --- |
| Age($\bar{x}\pm s$,years) |  | 48.67$\pm$9.12 | 52.67$\pm$9.74 |
| Male, n(%) |  | 14 (47) | 12 (40) |
| Atrophic, n(%) | absent | 30 (100) | 0 (0) |
|  | mild | 0 (0) | 14 (47) |
|  | moderate | 0 (0) | 11 (37) |
|  | severe | 0 (0) | 5 (17) |
| Interestnal metaplasia, n(%) | absent | 30 (100) | 12 (40) |
|  | mild | 0 (0) | 9 (30) |
|  | moderate | 0 (0) | 8 (27) |
|  | severe | 0 (0) | 1 (3) |
| Dysplasia, n(%) | absent | 30 (100) | 22 (73) |
|  | Light-median | 0 (0) | 8 (27) |
| Chronic inflammation, n(%) | No activity | 1 (3) | 0 (0) |
|  | mild | 19 (63) | 11 (37) |
|  | moderate | 10 (33) | 14 (47) |
|  | severe | 0 (0) | 5 (17) |
| Active inflammation, n(%) | No activity | 16 (53) | 24 (80) |
|  | mild | 14 (46.7) | 3 (10) |
|  | moderate | 0 (0) | 3 (10) |
|  | severe | 0 (0) | 0 (0) |

Table S3. Summary of sequencing data for each sample pools.

| Sample name | Sequence type | Raw tag count | Clean tag count | Mapped tag count | Percentage  (%) |
| --- | --- | --- | --- | --- | --- |
| CAG1 | SE50 | 29,131,714 | 22,441,854 | 19,239,403 | 66.04 |
| CAG2 | SE50 | 40,196,862 | 20,553,175 | 17,771,574 | 44.21 |
| CAG3 | SE50 | 30,803,352 | 24,933,911 | 20,977,948 | 68.10 |
| CNAG1 | SE50 | 41,623,105 | 19,055,122 | 13,951,302 | 33.52 |
| CNAG2 | SE50 | 36,129,051 | 29,236,146 | 17,510,955 | 48.47 |
| CNAG3 | SE50 | 35,509,180 | 30,114,801 | 25,416,856 | 71.58 |

Percentage(%)=Mapped tag count/Raw tag count

Table S4. Readnum of 220 miRNAs for each sample pools.

| miRNA id | CAG1 | CAG2 | CAG3 | CNAG1 | CNAG2 | CNAG3 | Description |
| --- | --- | --- | --- | --- | --- | --- | --- |
| hsa-miR-148a-3p | 1908809 | 1405407 | 1835968 | 1054084 | 610365 | 4140049 | mir-148 |
| hsa-miR-122-3p | 101580 | 168932 | 8251130 | 730336 | 98742 | 1131587 | mir-122 |
| hsa-miR-486-3p | 1770591 | 784865 | 794402 | 1653936 | 380472 | 2235387 | mir-486 |
| hsa-miR-451a | 743014 | 287424 | 667707 | 386514 | 983407 | 2929636 | mir-451 |
| hsa-miR-122-5p | 959300 | 2024398 | 1306496 | 41063 | 285397 | 1788271 | mir-122 |
| hsa-miR-3591-3p | 1399898 | 3129612 | 523078 | 264926 | 14925 | 156550 | mir-122 |
| hsa-miR-486-5p | 296390 | 232411 | 1227300 | 127488 | 274201 | 1608426 | mir-486 |
| hsa-miR-151a-3p | 597753 | 211730 | 430341 | 480548 | 208674 | 1356171 | mir-28 |
| hsa-miR-92a-3p | 79059 | 85521 | 205089 | 114734 | 124190 | 449321 | mir-25 |
| hsa-miR-320a | 197367 | 68577 | 286774 | 108454 | 48574 | 278722 | mir-320 |
| hsa-let-7b-5p | 205320 | 48536 | 125711 | 56486 | 21733 | 396840 | let-7 |
| hsa-let-7b-3p | 50196 | 38462 | 190615 | 110623 | 63406 | 118364 | let-7 |
| hsa-miR-25-3p | 63231 | 53262 | 106240 | 81381 | 79972 | 219866 | mir-25 |
| hsa-let-7a-3p | 69276 | 29849 | 105272 | 1590 | 28815 | 340271 | let-7 |
| hsa-miR-423-3p | 174930 | 5984 | 165206 | 5346 | 28144 | 39885 | mir-423 |
| hsa-let-7i-5p | 50381 | 31135 | 55308 | 52166 | 40148 | 167037 | let-7 |
| hsa-let-7f-5p | 1236 | 1932 | 38261 | 13913 | 2119 | 263222 | let-7 |
| hsa-miR-10b-5p | 69827 | 9799 | 29164 | 30839 | 50701 | 150731 | mir-10 |
| hsa-let-7i-3p | 34709 | 18017 | 10567 | 21276 | 27761 | 179367 | let-7 |
| hsa-miR-7641 | 719 | 1057 | 603 | 1303 | 468928 | 1372 | mir-7641 |
| hsa-miR-3184-3p | 21491 | 16806 | 98818 | 161732 | 15227 | 24342 | undef |
| hsa-let-7f-2-3p | 62822 | 45479 | 5264 | 8396 | 16497 | 139144 | let-7 |
| hsa-let-7a-2-3p | 3016 | 31975 | 27916 | 35172 | 31123 | 9028 | let-7 |
| hsa-let-7f-1-3p | 1992 | 22616 | 24500 | 2723 | 32246 | 25351 | let-7 |
| hsa-miR-1246 | 16605 | 127973 | 23179 | 12088 | 9310 | 27555 | mir-1246 |
| hsa-miR-423-5p | 36626 | 28742 | 9049 | 13187 | 50161 | 180342 | mir-423 |
| hsa-miR-10b-3p | 5662 | 32894 | 106606 | 7125 | 7073 | 61110 | mir-10 |
| hsa-miR-30d-5p | 30916 | 4530 | 67958 | 21695 | 17909 | 119932 | mir-30 |
| hsa-let-7a-5p | 19247 | 19782 | 4126 | 10492 | 2770 | 129139 | let-7 |
| hsa-miR-140-3p | 25163 | 19868 | 22622 | 55670 | 20571 | 92519 | mir-140 |
| hsa-miR-375 | 95324 | 16049 | 36390 | 16835 | 5766 | 70066 | mir-375 |
| novel_mir363 | 26600 | 20721 | 24599 | 13993 | 9972 | 45535 | chr16 |
| hsa-miR-22-3p | 35744 | 10978 | 59745 | 12609 | 14493 | 78317 | mir-22 |
| hsa-miR-378a-3p | 37425 | 29745 | 51066 | 17442 | 9808 | 49865 | mir-378 |
| hsa-miR-99a-5p | 67818 | 16745 | 85197 | 19775 | 690 | 79995 | mir-10 |
| hsa-miR-30d-3p | 14965 | 36087 | 45518 | 20704 | 11579 | 44968 | mir-30 |
| hsa-miR-143-3p | 26590 | 8950 | 27087 | 15285 | 10706 | 74665 | mir-143 |
| hsa-miR-126-3p | 13802 | 9251 | 23040 | 5545 | 15554 | 43882 | mir-126 |
| hsa-miR-10a-5p | 41554 | 14152 | 11980 | 4854 | 19238 | 63645 | mir-10 |
| hsa-miR-99a-3p | 6219 | 26141 | 25944 | 2932 | 14821 | 13886 | mir-10 |
| hsa-miR-192-5p | 5205 | 42424 | 89363 | 6565 | 3672 | 36945 | mir-192 |
| hsa-miR-10a-3p | 6228 | 10507 | 48477 | 15824 | 3625 | 30099 | mir-10 |
| hsa-miR-101-3p | 9935 | 5029 | 13274 | 17863 | 14222 | 75162 | mir-101 |
| hsa-miR-185-5p | 2144 | 18428 | 3302 | 11596 | 2070 | 58276 | mir-185 |
| hsa-miR-185-3p | 29501 | 4704 | 16286 | 3359 | 7374 | 19990 | mir-185 |
| novel_mir1 | 14229 | 18856 | 13849 | 7398 | 9523 | 16376 | chr7 |
| hsa-let-7g-3p | 11317 | 9547 | 5537 | 5793 | 5720 | 21662 | let-7 |
| hsa-miR-3184-5p | 5290 | 8896 | 21632 | 14852 | 231 | 28081 | undef |
| hsa-miR-106b-3p | 12288 | 5002 | 9606 | 11991 | 6778 | 24860 | mir-17 |
| hsa-miR-3615 | 10075 | 6312 | 13646 | 8947 | 4474 | 23293 | mir-3615 |
| hsa-miR-148b-3p | 14930 | 3557 | 7794 | 8593 | 5020 | 34992 | mir-148 |
| hsa-miR-192-3p | 8046 | 17247 | 13807 | 1508 | 1020 | 24346 | mir-192 |
| hsa-let-7d-3p | 7968 | 5917 | 15247 | 10819 | 4612 | 19409 | let-7 |
| hsa-miR-186-5p | 8291 | 2800 | 1151 | 564 | 7464 | 51987 | mir-186 |
| hsa-miR-3158-3p | 16993 | 459 | 1150 | 10860 | 530 | 25407 | mir-3158 |
| hsa-let-7g-5p | 475 | 2258 | 6914 | 2998 | 5844 | 48034 | let-7 |
| hsa-miR-128-3p | 7106 | 6390 | 8191 | 9710 | 3242 | 19433 | mir-128 |
| hsa-miR-146a-3p | 14747 | 4165 | 7403 | 4010 | 373 | 15843 | mir-146 |
| hsa-miR-21-3p | 3082 | 6585 | 2385 | 2424 | 4535 | 27188 | mir-21 |
| hsa-miR-320b | 17481 | 1983 | 4315 | 6090 | 556 | 8614 | mir-320 |
| hsa-let-7c-3p | 1611 | 8062 | 13500 | 1015 | 371 | 27282 | let-7 |
| hsa-miR-28-3p | 6130 | 5949 | 9254 | 5743 | 2213 | 11513 | mir-28 |
| hsa-miR-100-5p | 1295 | 4206 | 26715 | 1459 | 1957 | 13913 | mir-10 |
| hsa-miR-139-5p | 3454 | 446 | 6199 | 4156 | 4678 | 2575 | mir-139 |
| hsa-miR-21-5p | 3733 | 324 | 13046 | 6331 | 6394 | 13623 | mir-21 |
| hsa-miR-1290 | 2673 | 12595 | 3126 | 3187 | 2857 | 7849 | mir-1290 |
| hsa-miR-27b-3p | 6055 | 4283 | 7084 | 8227 | 3487 | 15564 | mir-27 |
| hsa-miR-99b-3p | 6846 | 1642 | 1009 | 4001 | 375 | 15809 | mir-10 |
| hsa-miR-16-2-3p | 7748 | 2130 | 5139 | 4776 | 3723 | 17718 | mir-15 |
| hsa-miR-100-3p | 7947 | 14197 | 7812 | 2817 | 1932 | 7300 | mir-10 |
| hsa-miR-27a-3p | 2290 | 1709 | 5231 | 1671 | 4102 | 21296 | mir-27 |
| hsa-miR-3158-5p | 580 | 5403 | 9989 | 638 | 5692 | 864 | mir-3158 |
| hsa-miR-99b-5p | 1675 | 3705 | 17061 | 1602 | 3461 | 3671 | mir-10 |
| hsa-miR-1307-3p | 11550 | 3522 | 3558 | 5887 | 1763 | 14581 | mir-1307 |
| hsa-miR-223-3p | 3638 | 1395 | 14378 | 1222 | 1440 | 15001 | mir-223 |
| hsa-miR-146a-5p | 2900 | 1152 | 5816 | 3851 | 2578 | 14064 | mir-146 |
| hsa-miR-186-3p | 1132 | 330 | 7689 | 7721 | 642 | 4587 | mir-186 |
| hsa-miR-182-3p | 3171 | 2844 | 1343 | 2996 | 1667 | 13407 | mir-182 |
| hsa-miR-629-5p | 8361 | 3728 | 6686 | 630 | 593 | 13662 | mir-629 |
| hsa-miR-183-5p | 3298 | 1516 | 2473 | 5779 | 767 | 12268 | mir-183 |
| hsa-miR-222-3p | 3875 | 2295 | 4859 | 2042 | 2537 | 8615 | mir-221 |
| hsa-miR-223-5p | 6097 | 2974 | 590 | 3075 | 1678 | 2506 | mir-223 |
| hsa-miR-3074-5p | 590 | 3009 | 13854 | 2594 | 729 | 15670 | mir-3074 |
| hsa-miR-194-5p | 480 | 5770 | 518 | 128 | 3200 | 21351 | mir-194 |
| hsa-miR-30a-3p | 4289 | 2522 | 5969 | 2319 | 3991 | 7345 | mir-30 |
| hsa-miR-532-5p | 999 | 102 | 6796 | 1209 | 1404 | 14204 | mir-188 |
| hsa-miR-183-3p | 739 | 4224 | 1605 | 2438 | 4280 | 13209 | mir-183 |
| hsa-miR-425-3p | 2195 | 2450 | 3293 | 359 | 1730 | 6861 | mir-425 |
| hsa-miR-584-3p | 1539 | 2295 | 4382 | 1286 | 774 | 17690 | mir-584 |
| hsa-miR-3074-3p | 11022 | 3450 | 773 | 3155 | 4026 | 7914 | mir-3074 |
| hsa-miR-532-3p | 5771 | 4508 | 814 | 4441 | 5339 | 2352 | mir-188 |
| hsa-miR-24-3p | 3090 | 934 | 636 | 1760 | 1875 | 4908 | mir-24 |
| hsa-let-7c-5p | 1572 | 3422 | 4950 | 1103 | 2104 | 9496 | let-7 |
| hsa-miR-139-3p | 2287 | 4075 | 2222 | 1791 | 681 | 12355 | mir-139 |
| hsa-miR-146b-5p | 1677 | 4462 | 7342 | 1522 | 1647 | 6064 | mir-146 |
| hsa-miR-16-1-3p | 32 | 3275 | 2965 | 965 | 6637 | 12852 | mir-15 |
| hsa-miR-26a-1-3p | 612 | 166 | 7562 | 524 | 579 | 12981 | mir-26 |
| hsa-miR-2110 | 4530 | 2329 | 3602 | 1262 | 846 | 4454 | mir-2110 |
| hsa-miR-744-3p | 3736 | 838 | 4560 | 3366 | 639 | 8359 | mir-744 |
| hsa-let-7d-5p | 153 | 878 | 4137 | 781 | 397 | 11266 | let-7 |
| hsa-miR-30e-3p | 2324 | 3061 | 3931 | 2254 | 2162 | 10433 | mir-30 |
| hsa-miR-7706 | 4110 | 2147 | 2865 | 4431 | 1221 | 5454 | undef |
| hsa-miR-629-3p | 2148 | 1377 | 1427 | 4904 | 1545 | 5594 | mir-629 |
| hsa-miR-16-5p | 102 | 141 | 1066 | 2187 | 808 | 19972 | mir-15 |
| novel_mir1399 | 3401 | 3080 | 3878 | 1246 | 601 | 3599 | chr17 |
| hsa-miR-182-5p | 1608 | 3690 | 1305 | 1939 | 2466 | 5363 | mir-182 |
| hsa-miR-584-5p | 1968 | 1545 | 1457 | 1795 | 2727 | 4378 | mir-584 |
| hsa-miR-26a-5p | 1722 | 867 | 700 | 209 | 446 | 2630 | mir-26 |
| hsa-miR-409-3p | 3034 | 1043 | 2874 | 1459 | 696 | 4695 | mir-154 |
| hsa-miR-30a-5p | 963 | 2013 | 6934 | 891 | 1310 | 5341 | mir-30 |
| hsa-miR-215-3p | 6619 | 217 | 1719 | 147 | 234 | 11096 | mir-192 |
| hsa-miR-194-3p | 8165 | 941 | 7710 | 3177 | 98 | 798 | mir-194 |
| hsa-miR-125a-3p | 1815 | 2280 | 2824 | 318 | 479 | 1059 | mir-10 |
| hsa-miR-181a-2-3p | 2993 | 1441 | 7809 | 568 | 1680 | 2435 | mir-181 |
| hsa-miR-363-3p | 896 | 653 | 2155 | 641 | 2355 | 7828 | mir-363 |
| hsa-miR-127-3p | 2128 | 869 | 1465 | 1654 | 471 | 2623 | mir-127 |
| hsa-miR-150-3p | 3339 | 751 | 3449 | 1214 | 1743 | 4257 | mir-150 |
| hsa-miR-425-5p | 2408 | 661 | 1754 | 3223 | 2253 | 3095 | mir-425 |
| hsa-miR-941 | 1669 | 1265 | 2297 | 2530 | 444 | 2958 | mir-941 |
| hsa-miR-20a-5p | 16 | 220 | 14 | 59 | 2578 | 10954 | mir-17 |
| hsa-miR-26a-2-3p | 56 | 4441 | 567 | 2280 | 5186 | 4159 | mir-26 |
| hsa-miR-146b-3p | 1916 | 4367 | 917 | 67 | 1902 | 5106 | mir-146 |
| hsa-miR-320c | 395 | 832 | 7321 | 1743 | 1269 | 1047 | mir-320 |
| hsa-miR-744-5p | 817 | 2461 | 986 | 656 | 502 | 2343 | mir-744 |
| hsa-miR-221-3p | 904 | 1153 | 2097 | 908 | 922 | 4037 | mir-221 |
| hsa-miR-1180-3p | 1477 | 1075 | 1956 | 880 | 716 | 3067 | mir-1180 |
| hsa-miR-501-3p | 1166 | 1161 | 1659 | 1983 | 895 | 2972 | mir-500 |
| hsa-miR-191-5p | 1139 | 732 | 1269 | 1707 | 497 | 3777 | mir-191 |
| hsa-miR-191-3p | 630 | 3140 | 1178 | 272 | 374 | 2820 | mir-191 |
| hsa-miR-142-3p | 1073 | 1112 | 1530 | 587 | 406 | 8573 | mir-142 |
| hsa-miR-361-3p | 988 | 1357 | 2309 | 1017 | 579 | 4945 | mir-361 |
| hsa-miR-1273a | 792 | 1466 | 3654 | 542 | 632 | 1393 | mir-1273 |
| hsa-miR-23a-3p | 1835 | 705 | 2246 | 234 | 1276 | 2582 | mir-23 |
| hsa-miR-152-3p | 868 | 2285 | 1016 | 1121 | 314 | 2877 | mir-148 |
| hsa-miR-664a-3p | 1544 | 244 | 2414 | 1219 | 682 | 1576 | mir-664 |
| hsa-miR-181a-3p | 330 | 1964 | 456 | 22 | 239 | 3004 | mir-181 |
| hsa-miR-215-5p | 823 | 820 | 986 | 2718 | 1597 | 3155 | mir-192 |
| hsa-miR-576-3p | 1343 | 456 | 843 | 733 | 551 | 3517 | mir-576 |
| hsa-miR-7-1-3p | 552 | 515 | 878 | 2115 | 561 | 3434 | mir-7 |
| hsa-miR-125a-5p | 966 | 287 | 1340 | 1371 | 944 | 3744 | mir-10 |
| hsa-miR-484 | 1245 | 914 | 1990 | 779 | 1045 | 2114 | mir-484 |
| hsa-miR-103b | 248 | 1521 | 353 | 360 | 89 | 869 | mir-103 |
| hsa-miR-193a-3p | 1820 | 2062 | 2092 | 776 | 853 | 1642 | mir-193 |
| novel_mir607 | 711 | 561 | 1477 | 306 | 710 | 4422 | chr1 |
| hsa-miR-3529-3p | 1212 | 1990 | 44 | 434 | 231 | 3554 | mir-3529 |
| hsa-miR-17-3p | 20 | 230 | 86 | 1254 | 686 | 3960 | mir-17 |
| hsa-miR-7-2-3p | 963 | 481 | 619 | 78 | 158 | 2778 | mir-7 |
| hsa-miR-4732-3p | 1136 | 243 | 961 | 1187 | 616 | 3163 | undef |
| hsa-miR-181a-5p | 61 | 88 | 135 | 764 | 1025 | 6170 | mir-181 |
| hsa-miR-328-3p | 1191 | 775 | 1450 | 935 | 149 | 1832 | mir-328 |
| hsa-miR-26b-3p | 38 | 608 | 1052 | 12 | 872 | 4018 | mir-26 |
| hsa-miR-206 | 1683 | 432 | 2438 | 1652 | 310 | 1796 | mir-1 |
| hsa-miR-134-5p | 776 | 657 | 140 | 778 | 65 | 1950 | mir-134 |
| hsa-miR-193a-5p | 2074 | 306 | 1427 | 592 | 31 | 832 | mir-193 |
| hsa-miR-26b-5p | 619 | 954 | 162 | 289 | 728 | 3127 | mir-26 |
| hsa-miR-664a-5p | 1118 | 910 | 838 | 36 | 162 | 3776 | mir-664 |
| hsa-miR-15b-3p | 1049 | 427 | 857 | 222 | 468 | 5910 | mir-15 |
| hsa-miR-19b-3p | 649 | 331 | 1119 | 361 | 1519 | 3874 | mir-19 |
| hsa-miR-4508 | 514 | 583 | 856 | 558 | 699 | 1577 | undef |
| hsa-miR-483-3p | 371 | 5036 | 1516 | 392 | 328 | 234 | mir-483 |
| hsa-miR-30e-5p | 244 | 806 | 1329 | 712 | 940 | 2174 | mir-30 |
| hsa-miR-197-3p | 593 | 551 | 730 | 375 | 363 | 1975 | mir-197 |
| hsa-miR-370-3p | 646 | 339 | 414 | 446 | 57 | 1095 | mir-370 |
| hsa-miR-125b-2-3p | 637 | 445 | 2328 | 484 | 83 | 1342 | mir-10 |
| hsa-miR-1285-3p | 631 | 557 | 780 | 974 | 132 | 1126 | mir-1285 |
| hsa-let-7e-5p | 187 | 644 | 1233 | 956 | 31 | 1466 | let-7 |
| hsa-miR-342-3p | 1140 | 105 | 2109 | 1644 | 326 | 710 | mir-342 |
| hsa-miR-15b-5p | 22 | 40 | 27 | 1184 | 1867 | 594 | mir-15 |
| hsa-miR-145-3p | 1259 | 338 | 1127 | 244 | 546 | 1526 | mir-145 |
| hsa-miR-1-3p | 490 | 350 | 117 | 13 | 460 | 1501 | mir-1 |
| hsa-miR-483-5p | 600 | 3563 | 383 | 193 | 10 | 272 | mir-483 |
| hsa-miR-7-5p | 852 | 40 | 13 | 117 | 655 | 771 | mir-7 |
| hsa-miR-134-3p | 875 | 27 | 1884 | 33 | 129 | 607 | mir-134 |
| hsa-miR-320d | 1870 | 141 | 1431 | 420 | 226 | 501 | mir-320 |
| novel_mir780 | 410 | 467 | 950 | 155 | 816 | 405 | chr5 |
| hsa-miR-6842-3p | 623 | 228 | 318 | 287 | 172 | 1763 | undef |
| hsa-miR-382-5p | 665 | 133 | 158 | 380 | 103 | 868 | mir-154 |
| hsa-miR-511-3p | 873 | 45 | 1832 | 775 | 13 | 178 | mir-506 |
| hsa-miR-671-3p | 634 | 509 | 698 | 339 | 57 | 427 | mir-671 |
| hsa-let-7e-3p | 763 | 335 | 404 | 77 | 576 | 1435 | let-7 |
| hsa-miR-582-3p | 104 | 171 | 393 | 334 | 297 | 2074 | mir-582 |
| hsa-miR-503-5p | 500 | 447 | 289 | 156 | 156 | 1401 | mir-503 |
| hsa-miR-125b-5p | 1605 | 443 | 109 | 66 | 242 | 583 | mir-10 |
| hsa-miR-378c | 571 | 142 | 835 | 311 | 23 | 1095 | undef |
| hsa-miR-625-3p | 428 | 481 | 673 | 344 | 262 | 940 | mir-625 |
| hsa-miR-150-5p | 23 | 59 | 717 | 257 | 241 | 2406 | mir-150 |
| hsa-miR-500a-3p | 452 | 737 | 199 | 221 | 291 | 1312 | mir-500 |
| hsa-miR-432-5p | 675 | 33 | 523 | 708 | 195 | 918 | mir-432 |
| hsa-miR-92b-3p | 674 | 199 | 502 | 201 | 202 | 856 | mir-25 |
| hsa-miR-93-5p | 1167 | 733 | 693 | 62 | 154 | 696 | mir-17 |
| hsa-miR-3929 | 191 | 355 | 348 | 733 | 124 | 133 | undef |
| hsa-miR-4732-5p | 24 | 216 | 1740 | 437 | 279 | 826 | undef |
| hsa-miR-1306-3p | 533 | 258 | 220 | 715 | 84 | 797 | mir-1306 |
| hsa-miR-30c-1-3p | 54 | 71 | 1277 | 257 | 298 | 1014 | mir-30 |
| hsa-miR-1273f | 104 | 420 | 778 | 179 | 72 | 75 | mir-1273 |
| hsa-miR-199a-3p | 59 | 170 | 891 | 198 | 227 | 1196 | mir-199 |
| hsa-miR-29a-3p | 181 | 217 | 997 | 309 | 476 | 811 | mir-29 |
| hsa-miR-17-5p | 583 | 489 | 752 | 26 | 347 | 359 | mir-17 |
| hsa-miR-432-3p | 398 | 155 | 309 | 233 | 303 | 1038 | mir-432 |
| hsa-miR-193b-5p | 1289 | 211 | 434 | 16 | 118 | 324 | mir-193 |
| hsa-miR-23b-3p | 268 | 428 | 869 | 11 | 274 | 925 | mir-23 |
| hsa-miR-379-3p | 34 | 91 | 206 | 18 | 10 | 290 | mir-379 |
| hsa-miR-98-3p | 363 | 150 | 281 | 230 | 152 | 862 | let-7 |
| hsa-miR-195-3p | 606 | 274 | 483 | 146 | 189 | 181 | mir-15 |
| hsa-miR-193b-3p | 456 | 260 | 760 | 175 | 42 | 353 | mir-193 |
| hsa-miR-98-5p | 168 | 66 | 437 | 52 | 85 | 713 | let-7 |
| hsa-miR-339-3p | 482 | 187 | 345 | 387 | 65 | 143 | mir-339 |
| hsa-miR-181b-3p | 435 | 208 | 371 | 241 | 58 | 220 | mir-181 |
| novel_mir53 | 173 | 102 | 475 | 321 | 91 | 182 | chr7 |
| hsa-miR-340-3p | 23 | 71 | 24 | 320 | 86 | 655 | mir-340 |
| hsa-miR-181b-5p | 18 | 174 | 254 | 428 | 67 | 122 | mir-181 |
| hsa-miR-502-3p | 192 | 90 | 121 | 15 | 76 | 153 | mir-500 |
| hsa-miR-382-3p | 38 | 36 | 474 | 38 | 80 | 496 | mir-154 |
| hsa-miR-378d | 41 | 186 | 619 | 11 | 74 | 150 | mir-378 |
| hsa-miR-155-3p | 18 | 12 | 86 | 155 | 249 | 299 | mir-155 |
| hsa-miR-4429 | 224 | 39 | 177 | 119 | 46 | 251 | mir-4429 |
| hsa-miR-378i | 19 | 85 | 139 | 10 | 17 | 254 | undef |
| hsa-miR-378f | 43 | 74 | 134 | 16 | 18 | 93 | undef |
| novel_mir503 | 43 | 15 | 28 | 13 | 14 | 50 | chr4 |
| hsa-miR-6131 | 19 | 19 | 32 | 24 | 22 | 19 | mir-6131 |
